# Comparison of the Mortality Prediction Value of Soluble Urokinase Plasminogen Activator Receptor (suPAR) in COVID-19 and Sepsis

**DOI:** 10.3390/diagnostics12051261

**Published:** 2022-05-18

**Authors:** Alice G. Vassiliou, Alexandros Zacharis, Charikleia S. Vrettou, Chrysi Keskinidou, Edison Jahaj, Zafeiria Mastora, Stylianos E. Orfanos, Ioanna Dimopoulou, Anastasia Kotanidou

**Affiliations:** First Department of Critical Care Medicine & Pulmonary Services, School of Medicine, National and Kapodistrian University of Athens, Evangelismos Hospital, 106 76 Athens, Greece; alexandroszacharis5@gmail.com (A.Z.); vrettou@hotmail.com (C.S.V.); chrysakes29@gmail.com (C.K.); edison.jahaj@gmail.com (E.J.); zafimast@yahoo.gr (Z.M.); sorfanos@med.uoa.gr (S.E.O.); idimo@otenet.gr (I.D.)

**Keywords:** suPAR, COVID-19, ICU, mortality, triage, sepsis

## Abstract

In the last years, biomarkers of infection, such as the soluble urokinase plasminogen activator receptor (suPAR), have been extensively studied as potential diagnostic and prognostic biomarkers in the intensive care unit (ICU). In this study, we investigated whether this biomarker can be used in COVID-19 and non-COVID-19 septic patients for mortality prediction. Serum suPAR levels were measured in 79 non-COVID-19 critically ill patients upon sepsis (within 6 h), and on admission in 95 COVID-19 patients (66 critical and 29 moderate/severe). The non-COVID-19 septic patients were matched for age, sex, and disease severity, while the site of infection was the respiratory system. On admission, COVID-19 patients presented with higher suPAR levels, compared to non-COVID-19 septic patients (*p* < 0.01). More importantly, suPAR measured upon sepsis could not differentiate survivors from non-survivors (*p* > 0.05), as opposed to suPAR measured on admission in COVID-19 survivors and non-survivors (*p* < 0.0001). By the generated ROC curve, the prognostic value of suPAR in COVID-19 was 0.81, at a cut-off value of 6.3 ng/mL (*p* < 0.0001). suPAR measured early (within 24 h) after hospital admission seems like a specific and sensitive mortality risk predictor in COVID-19 patients. On the contrary, suPAR measured at sepsis diagnosis in non-COVID-19 critically ill patients, does not seem to be a prognostic factor of mortality.

## 1. Introduction

Since endothelial damage was recognized as an important pathobiological mechanism involved in COVID-19 [1], sepsis biomarkers have been shown to be relative in this disease [2,3,4,5]. Apart from the families of cell adhesion molecules, various receptor biomarkers implicated in sepsis, have also been shown elevated in COVID-19 [6].

The urokinase plasminogen activator (uPA) system is central to a spectrum of biological processes, including inflammation, fibrinolysis, cell proliferation, migration, and adhesion [7]. The binding of uPA to its receptor (uPAR) results in the conversion of plasminogen to plasmin via a proteolytic cascade [8]. Proteolytic cleavage of uPAR releases its soluble form, suPAR. suPAR was first identified in 1985 as a cellular binding site for urokinase [9], and since has been investigated as a potential prognostic marker in the intensive care unit (ICU). suPAR has been suggested to reflect the activation status of the immune system, rather than exerting inflammatory actions [10]. It is considered a pro-inflammatory biomarker associated with immune activation and fibrinolysis inhibition; its levels have been found to increase in several systemic diseases, including sepsis, cardiovascular disease, cancer, autoimmune conditions, kidney disease, and other organ failures [11]. Hence, it is not considered a disease-specific diagnostic marker. The study by Corban and colleagues [12] provided evidence for the link between fibrinolytic and inflammatory pathways and endothelial dysfunction. However, whether such biomarkers, including suPAR, can cause endothelial dysfunction or are only associated with it is still unknown.

In a recent study, we demonstrated that suPAR is useful in predicting mortality in critically ill COVID-19 patients [5]. Recently, a distinctive biomarker motif of endotheliopathy was shown for COVID-19 and septic syndromes [13]. Hence, in the present study, we aimed to characterize suPAR’s prognostic ability in COVID-19 pneumonia compared to sepsis arising from the respiratory tract.

## 2. Materials and Methods

This observational, single-center study included 95 consecutive COVID-19 patients admitted either in the ICU (N = 66, critically ill COVID-19 patients), or the ward (N = 29, moderate/severe patients), and 79 consecutive non-COVID-19, critically ill septic patients, admitted to the general, multi-disciplinary ICU of Evangelismos Hospital. Septic patients were matched for age, sex, and critical illness severity, while the source of sepsis was the respiratory tract. Septic patients with the site of infection at the abdomen (N = 1), the central nervous system (CNS) (N = 1), or the bloodstream (N = 6) were excluded from further analyses. SARS-CoV-2 infection was diagnosed by real-time reverse transcription PCR (RT-PCR) in nasopharyngeal swabs. Sepsis was defined as a life-threatening organ dysfunction caused by a dysregulated host response to infection, according to the third consensus definition for sepsis and septic shock [14]. All patients included in the study had pneumonia with respiratory failure, while all ICU patients and 19/29 (66%) of the ward patients fulfilled the Berlin criteria for acute respiratory distress syndrome (ARDS) [15]. The study was approved by the Hospital’s Research Ethics Committee (129/19-3-2020), and all procedures carried out on patients were in compliance with the Helsinki Declaration. Informed written consent was obtained from all patients’ next-of-kin.

The critically ill COVID-19 patients were immediately hospitalized in the ICU after the Emergency Department (ED). Following study enrolment, demographic characteristics, comorbidities, symptoms, vital signs, and laboratory findings were recorded. Acute physiology and chronic health evaluation (APACHE II) and sequential organ failure assessment (SOFA) score were calculated on ICU admission. The outcome was defined as overall mortality.

Four milliliters (4 mL) of venous blood were collected within the first 24 h post hospital admission in the COVID-19 patients, and within 6 h from sepsis diagnosis in the non-COVID-19 patients. Serum was drawn in BD Vacutainer^®^ Plus Plastic Serum Tubes. Serum was collected, portioned into 0.5 mL aliquots, and stored at −80 °C until used. suPAR was measured by enzyme-linked immunosorbent assay (ELISA) (R & D Systems Inc., Minneapolis, MN, USA).

Data are given as individual values N (%), mean ± standard deviation (SD), or median with interquartile range (IQR), accordingly. Comparisons were performed by the *t*-test, the non-parametric Mann-Whitney test, the chi-square test, or one way ANOVA, as appropriate. Correlations were performed by Spearman’s correlation coefficient. Receiver operating characteristic (ROC) curves were plotted using overall ICU mortality (or hospital mortality for the ward patients) as the classification variable and suPAR levels as prognostic variables. The optimal cut-off value for predicting mortality was calculated as the point with the greatest combined sensitivity and specificity. This value was then used to divide the patients into two groups, higher or lower than the ROC curve generated cut-off value, to perform Kaplan-Meier analysis for survival probability estimation; the log-rank test for a two-group comparison was used. The analyses were performed with IBM SPSS statistical package, version 22.0 (IBM Software Group, Armonk, NY, USA), and GraphPad Prism, version 8.0 (GraphPad Software, San Diego, CA, USA). Statistical significance was set at *p* < 0.05.

## 3. Results

### 3.1. Patient Characteristics

The demographics of the three patient groups are shown in Table 1. The groups did not differ in terms of age and sex. The non-COVID-19 septic patients had fewer comorbidities as expected, since this group included 43% trauma patients. Thirty-four percent (34%) of the patients had been subjected to emergent surgery, while 23% of the patients suffered from mainly CNS-related pathologies. The two ICU groups had a comparable disease severity. The site of infection in the septic patients was the respiratory system, and in 69 of them (87.3%) due to a Gram-negative bacterium, while in the remaining seven by a Gram-positive bacterium. Sepsis occurred between days 4–11 of ICU stay (median seven days). Twenty-six patients (33%) also developed septic shock later on during their ICU stay. The ICU COVID-19 group had a higher mortality, whereas the ICU non-COVID-19 septic group had higher length of stay and mechanical ventilation duration. Twenty-nine (44%) of ICU COVID-19 patients from the second wave received dexamethasone, as per guidelines; it should be noted that in a previous study we demonstrated that dexamethasone lowered suPAR levels, however not in a statistically significant manner [5].

### 3.2. Comparative Analysis of suPAR in COVID-19 and ICU-Acquired Sepsis

**Figure 1:**
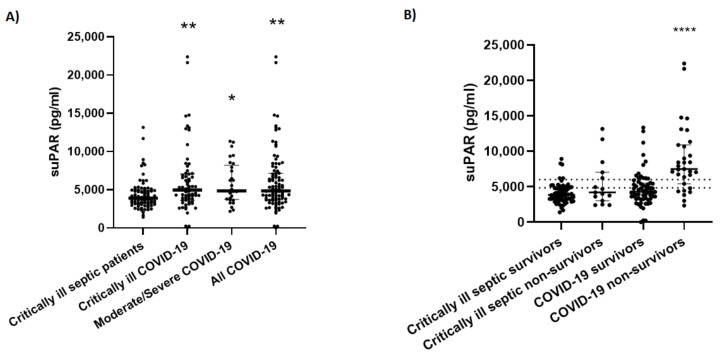
suPAR levels in COVID-19 and sepsis. (**A**) suPAR levels were measured on admission (within 24 h) in 66 critically ill and 29 moderate/severe COVID-19 patients, and 79 critically ill non-COVID-19 septic patients (within 6 h from sepsis diagnosis); (**B**) The COVID-19 and non-COVID-19 septic patients were divided in survivors and non-survivors, and their suPAR levels were compared. Horizontal lines, medians of the two groups. The groups were compared by ANOVA followed by Kruskal-Wallis (panel A, *p*-values against the septic group), or Mann-Whitney (panel B, between survivors and non-survivors within each group). * *p* < 0.05, ** *p* < 0.01, **** *p* < 0.0001. suPAR = soluble urokinase-type plasminogen activator receptor.

Both critical and moderate/severe COVID-19 patients had elevated suPAR levels compared to the ICU non-COVID-19 septic patients [4.95 (3.63–7.07) ng/mL and 4.84 (3.72–8.18 ng/mL), respectively vs. 3.90 (3.17–4.94 ng/mL); *p* < 0.05; Figure 1A]. Of interest, suPAR measured at the time of sepsis diagnosis could not differentiate survivors from non-survivors [3.85 (3.19–4.87) ng/mL vs. 4.19 (3.04–7.03) ng/mL; *p* = 0.3; Figure 1B], as opposed to suPAR measured on admission in COVID-19 patients [4.28 (3.31–5.52) ng/mL vs. 7.50 (5.42–10.89) ng/mL; *p* < 0.0001]. 

In the COVID-19 patients, suPAR positively correlated with the disease severity markers lactate dehydrogenase (LDH) (r_s_ = 0.46, *p* < 0.0001), D-dimers (r_s_ = 0.44, *p* < 0.0001), ferritin (r_s_ = 0.33, *p* = 0.009), and the APACHE II score (in the critically ill subpopulation) (r_s_ = 0.29, *p* = 0.02). Moreover, suPAR levels positively correlated with C-reactive protein (CRP) (r_s_ = 0.24, *p* = 0.02) and age (r_s_ = 0.38, *p* < 0.0001). Furthermore, the critically ill patients who were subsequently intubated also had higher admission suPAR levels, and these levels also correlated with prolonged mechanical ventilation duration (r_s_ = 0.22, *p* = 0.03). In the non-COVID-19 septic patients, suPAR levels positively correlated with age (r_s_ = 0.39, 0 < 0.0001), and the APACHE II score (r_s_ = 0.27, *p* = 0.02).

**Figure 2:**
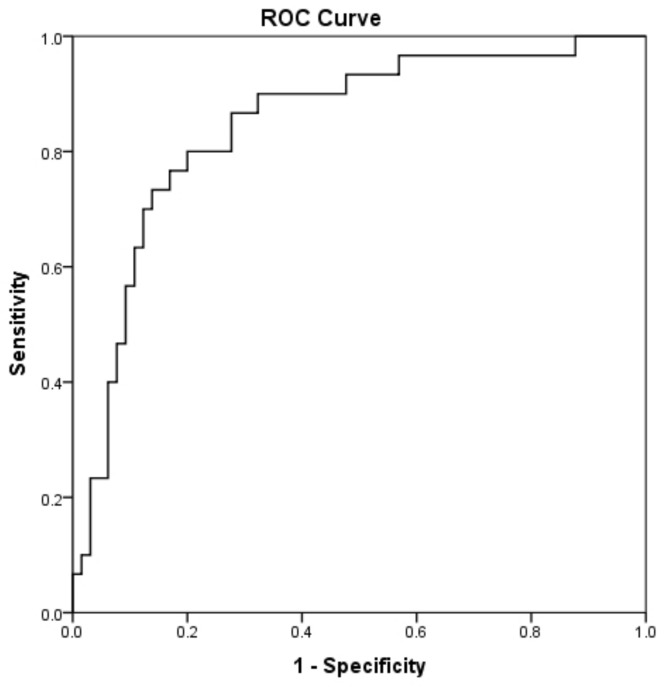
Receiver operating characteristic (ROC) curve analysis. A ROC curve was generated to determine the prognostic accuracy of suPAR on hospital admission (within 24 h). suPAR gave an AUC of 0.81 (0.71–0.91), *p* < 0.0001, at a cut-off value of 6.31 ng/mL, with greatest combined sensitivity of 74.2% and specificity 85.9% suPAR = soluble urokinase-type plasminogen activator receptor.

A ROC curve was generated to determine the prognostic accuracy of suPAR in predicting mortality in COVID-19; the area under the curve (AUC) of suPAR levels was 0.81 (95% CI = 0.71–0.91), *p* < 0.0001; Figure 2. According to the ROC curve analysis, the optimal cut-off point for suPAR was 6.3 ng/mL, with a greatest combined sensitivity of 74.2% (95% CI = 56.8–86.3) and specificity of 85.9% (95% CI = 75.4–92.4). 

**Figure 3:**
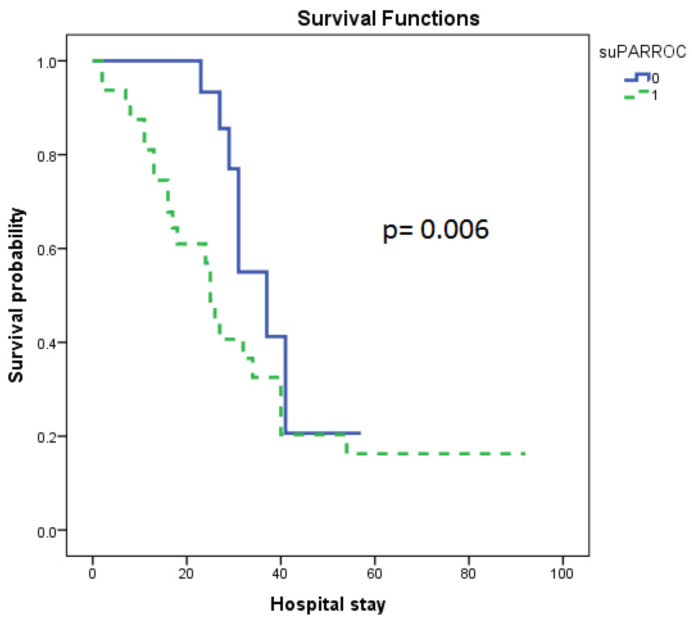
suPAR levels on admission and COVID-19 survival probability. suPAR levels were measured on hospital admission (within 24 h). The Kaplan-Meier method was used for survival probability estimation and the log-rank test for two-group comparison. The COVID-19 group was dichotomized above and below the cut-off value generated from the ROC curve (6.31 ng/mL). Dashed line ≥ cut-off value (high group, 1); solid line < cut-off value (low group, 0). The respective median time to mortality for the two aforementioned groups were 37 (23–51) days for the low group, and 25 (22–28) days for the high group (Log-Rank test, *p* = 0.006). suPAR = soluble urokinase-type plasminogen activator receptor.

Patients with lower (low group = 0) than the cut-off value generated from the ROC curve, were subsequently compared to the patients who had higher than the cut-off value (high group = 1). We found that the higher group was associated with a higher overall mortality in the Kaplan-Meier analysis. The respective median time to mortality for the aforementioned groups was 37 (23–51) days for the low group, and 25 (22–28) days for the high group (Log-Rank test, *p* = 0.006; Figure 3).

COVID-19 survivors and non-survivors differed only in terms of age, APACHE II scores (for the critically ill subpopulation), and suPAR levels on admission. The univariate and multivariate regression analyses showed that suPAR levels could be assumed as an independent predictor of mortality in COVID-19, in the presence of age [1.000 (1.000–1.001), *p* = 0.001]. Of note, a combined ROC curve of suPAR levels and age did not improve prognostic value.

## 4. Discussion

To our knowledge, this is the first report to compare the prognostic value of suPAR levels in critically ill COVID-19 and non-COVID-19 septic patients with respiratory infections in terms of mortality. Our results showed that in our cohorts, suPAR can differentiate COVID-19 patients who will not survive their illness, but not septic patients. 

In COVID-19, suPAR has been shown elevated in severe versus moderate disease, and furthermore, has been attributed a prognostic value in identifying patients with a poor prognosis, such as prolonged stay, or requirement of mechanical ventilation [16,17,18,19,20,21]. Very few studies have investigated its role in mortality [5,22,23]. We previously showed that critically ill COVID-19 patients who will not survive their disease have much higher ICU admission suPAR levels compared to survivors [5]. The prognostic ability of suPAR did not seem to be affected by the administration of dexamethasone. Similarly, Napolitano et al. proposed suPAR as a serum biomarker of clinical severity and outcome in patients who were hospitalized with COVID-19 [23]. They demonstrated that the non-survivor group exhibited higher levels of serum suPAR (4.5 ng/mL) than the survivor group (3.2 ng/mL), suggesting that suPAR may be predictive of survival. 

On the other hand, the utility of suPAR in diagnosing sepsis has been confirmed in many studies [24]. Its role, however, as a prognosticator of poor sepsis outcomes, and specifically in predicting mortality in the context of sepsis has arisen controversies. In a very recent study, it was shown that suPAR could predict early mortality among sepsis patients at a cut-off value of 13.4 ng/mL [25]. Furthermore, suPAR could be assumed a predictor of bad prognosis and poor survival in sepsis, seven days following admission [26]. High suPAR levels on admission in initially septic and non-septic patients were shown to be an independent predictor for ICU and 28-day mortality [27]. suPAR was shown to be stably elevated during the first week of treatment in the ICU, and could predict mortality in both sepsis and non-sepsis critically ill patients [28]. The authors suggested that the high suPAR concentrations upon ICU admission likely reflected activation of the immune system, prior to sepsis development. It is possible that the lower presence of comorbidities in our cohort may influence suPAR levels and affect its predictive ability in the septic ICU population.

Limitations of our study include its single-center nature, the moderate number of patients, and the low mortality rate (19%) in the non-COVID-19 critically ill septic patients. Furthermore, the COVID-19 group had a higher incidence of comorbidities, which might explain the higher mortality. However, the strengths were the well characterized non-COVID-19 patients, who on ICU admission were initially non-septic. Patients who developed sepsis within 24–48 h from ICU admission were excluded in order to exclude underlying sepsis. suPAR was measured in samples collected within 6 h from sepsis occurrence. We also matched our septic patients to the COVID-19 patients, based on age, critical illness severity, as expressed by the APACHE II and SOFA scores, and selected patients with a site of infection in the respiratory system.

## 5. Conclusions

Our results demonstrated the presence of high levels of circulating suPAR in COVID-19 compared to another severe inflammatory syndrome, namely sepsis, caused mostly by Gram-negative bacteria in the respiratory system. Furthermore, in COVID-19, yet not in sepsis, suPAR levels could differentiate survivors from non-survivors, with a good prognostic accuracy from the ROC curve generated. Combined with other biomarkers, a profile can be built for COVID-19 patients with a clear association with poor outcome prediction, exhibiting a distinctive pattern from septic syndromes.

## Figures and Tables

**Table 1:** Demographics and clinical characteristics of the patients.

Characteristics	ICU COVID-19	Moderate/Severe COVID-19	ICU Non-COVID-19 Septic Patients
Number of patients, N	66	29	79
Age (years), (mean ± SD)	64 ± 12	65 ± 19	60 ± 12
Sex, N (%)			
Male	51 (77.3)	21 (72.4)	55 (69.6)
Female	15 (22.7)	8 (27.6)	24 (30.4)
APACHE II, (mean ± SD)	15 ± 4	N/A	16 ± 5
SOFA, (mean ± SD)	6 ± 3	N/A	7 ± 3
Comorbidities, N (%)	50 (75.6) ****	21 (72.4) ****	34 (43.0)
Hypertension	30	12	24
Hyperlipidemia	17	6	5
Diabetes	9	3	6
Coronary artery disease	9	5	2
Diagnosis			
Medical	66 (100) ****	29 (100) ****	18 (22.8)
Surgical/Trauma	0 (0)	0 (0)	61 (77.2)
PaO_2_/FiO_2_ (mmHg), (mean ± SD)	184 ± 91 ****	284 ± 96	261 ± 116
White blood cell count (per μL), (median, IQR)	9980 (6520–11,890) **	4980 (4200–7613) ****	12,190 (9080–14,880)
CRP (mg/dL), (median, IQR)	11.5 (5.3–20.0)	13.2 (6.4–45.8) **	11.5 (7.1–15.1)
PCT (ng/mL), (median, IQR)	0.30 (0.11–1.30)	0.11 (0.05–0.30)	0.17 (0.10–0.52)
Inotropes mean dose (µg/kg/min), (median, IQR)	0.19 (0.09–0.30)	N/A	0.10 (0.00–0.23)
Lactate (mmol/L), (median, IQR)	1.5 (1.1–1.9)	1.1 (0.8–1.7)	1.4 (1.0–2.0)
WHO clinical progression scale (median, IQR)	7 (7–9)	5 (4–6)	N/A
Dexamethasone, N (%) ^†^	29 (43.9) ****	0 (0)	0 (0)
Endothelial markers suPAR (ng/mL), (median, IQR)	4.9 (3.6–7.1) **	4.8 (3.7–8.2) *	3.9 (3.2–4.9)
Outcomes			
Length of stay (days), (median, IQR)	18 (11–31) ****	13 (7–21) ****	31 (23–40)
Mechanical ventilation, N (%)	51 (77.3) ****	N/A	79 (100)
Duration of mechanical ventilation (days), (median, IQR)	10 (3–27) ****	N/A	21 (16–30)
Mortality	23 (34.8) *	8 (27.6)	15 (19.0)

* *p*-value < 0.05; ** *p*-value < 0.01; **** *p*-value < 0.0001 from the ICU non-COVID-19 septic patient group. ^†^ Twenty-nine patients were from the second wave and received dexamethasone, whereas 37 were recruited at the start of the pandemic, prior to the adoption of the dexamethasone administration guidelines. Data are expressed as number of patients (N), percentages of total related variable (%), or mean ± SD for normally distributed variables and median (IQR) for skewed data. The Kruskal-Wallis test or the chi-square test was used, as appropriate. Characteristics were measured within 24 h from admission in the COVID-19 patients, whereas in the ICU non-COVID-19 septic patients, the data given are at sepsis onset (within 6 h). Definition of abbreviations: APACHE = Acute physiology and chronic health evaluation; CRP = C-reactive protein; ICU = Intensive care unit; PCT = Procalcitonin; SOFA = Sequential organ failure assessment; suPAR = Soluble urokinase plasminogen activator receptor.

## Data Availability

Data available on request.
